# *Astragalus* and *Paeoniae* radix rubra extract inhibits liver fibrosis by modulating the transforming growth factor-β/Smad pathway in rats

**DOI:** 10.3892/mmr.2014.2868

**Published:** 2014-11-05

**Authors:** WEIJUAN HUANG, LIN LI, XIAOPENG TIAN, JINJIN YAN, XINZHENG YANG, XINLONG WANG, GUOZHEN LIAO, GENQUAN QIU

**Affiliations:** 1 Department of Scientific Research, Xi’an Medical College, Xi’an, Shaanxi 710061, P.R. China; 2 State Key Laboratory of Oncology in South China; Collaborative Innovation Center of Cancer Medicine, Sun Yat-sen University, Guangzhou, Guangdong 510060, P.R. China; 3 Department of Pharmacology, Xi’an Medical College, Xi’an, Shanxi 710061, P.R. China; 4 Department of Traditional Chinese Medicine, First Affiliated Hospital of Xi’an Jiao Tong University, Xi’an, Shanxi 710061, P.R. China

**Keywords:** *Astragalus* and *Paeoniae* radix rubra extract, transforming growth factor-β/Smad pathway, hepatic fibrosis, carbon tetrachloride

## Abstract

It has been previously demonstrated that *Astragalus* and *Paeoniae* radix rubra extract (APE) had a protective effect against liver fibrosis in mice. The present study aimed to investigate the hepatoprotective effect of APE on CCl_4_-induced hepatic fibrosis in rats. Liver fibrosis was induced in male Sprague-Dawley rats by intraperitoneal injection of 50% CCl_4_ twice a week for eight weeks. Organ coefficients, serum aspartate aminotransferase (AST), alanine aminotransferase (ALT), hexadecenoic acid (HA), laminin (LN), procollagen type III (PCIII), hydroxyproline (Hyp), glutathione (GSH-Px), malondialdehyde (MDA), superoxide dismutase (SOD) and transforming growth factor β1 (TGF-β1) levels were measured in rats with hepatic fibrosis. Histopathological changes in affected livers were studied using hematoxylin-eosin and Masson’s trichrome staining. The expression of transforming growth factor-β/Smad pathway proteins, α-smooth muscle actin (α-SMA), collagen I and collagen III was observed in fibrotic livers using western blot analysis. The present study observed significant reductions in serum levels of AST, ALT, HA, LN, PCIII and Hyp in APE-treated (2.6 and 5.2 g/kg) rats, indicating the significant hepatoprotective effects of APE. Furthermore, the depletion of GSH-Px and SOD, in addition to the accumulation of MDA in liver tissue was suppressed by APE (2.6 and 5.2 g/kg). Pathological assessment of CCl_4_-induced fibrotic livers revealed a significant reduction of liver injury and development of hepatic fibrosis in rats treated with APE (2.6 and 5.2 g/kg). Moreover, APE (2.6 and 5.2 g/kg) decreased the elevation of TGF-β1, α-SMA, collagen I and collagen III expression, inhibited Smad2/3 phosphorylation as well as elevated the expression of the TGF-β1 inhibitor Smad7. These results suggested that APE may protect against liver damage and inhibit the progression of CCl_4_-induced hepatic fibrosis. The mechanism of action of APE is hypothesized to proceed via scavenging free radicals, decreasing TGF-β1 levels and blocking of the TGF-β/Smad signaling pathway.

## Introduction

Hepatic fibrosis (HF) is recognized as one of the most prevalent types of liver disease. Biologically, HF is defined as the wound-healing process that occurs as a result of a wide range of inflammatory reactions in the liver (1–3). There are numerous enivronmental toxins for chronic liver disease, including cholestasis, circulatory disturbances, autoimmune and nutrition disorders, environmental toxins and the use of particular medicine; however, the two primary causes of liver fibrosis have been identified as infections caused by the hepatitis virus and alcoholism (4). Regardless of its origin, liver fibrosis is progressive and eventually leads to cirrhosis or hepatocellular carcinoma, ultimately resulting in organ failure and risk of mortality (5).

HF is characterized by the overabundant deposition of extracellular matrix (ECM) proteins, composed mainly of type I and type III collagen proteins. These excessive depositions disturb the normal structure of the hepatic lobule, resulting in misdirected blood flow through the liver, thereby impairing normal organ functioning. This is the most salient feature of liver cirrhosis (6).

Early in the progression of hepatic fibrosis, a potent, fibrinogenic cytokine, transforming growth factor-β (TGF-β), was demonstrated to be locally and systemically increased in response to acute as well as chronic liver injury (7). TGF-β has been reported to trigger the activation of hepatic stellate cells (HSCs), inducing their differentiation into fibroblasts. This transformation has been shown to be the principle determinant for the accumulation of extracellular matrix proteins (8,9). Activation of HSCs is therefore thought to be a key step in the progression of hepatic fibrosis, justifying their use as a major therapeutic target for the prevention and treatment of liver cirrhosis (10). Due to the connection between TGF-β and HSCs, therapeutic modalities that may inhibit or reverse the action of either in order to prevent the progression of hepatic fibrosis are the focus of present studies.

Recent studies indicated that hepatic fibrosis is a complex pathological process that involves various cytokines and numerous cell signaling pathways (11–14). TGF-β1 has been established as the crucial fibrogenic cytokine promoting liver fibrosis, due to its activation of HSCs via the TGF-β/Smad signaling pathway (15). In addition, it has been reported that fibrosis has a dynamic bidirectional nature (16,17); however, no effective therapies or medicine aimed at its reversal are currently available, making their development necessary and urgent.

*Astragalus* and *Paeoniae* radix rubra extract (APE) is composed primarily of paeoniflorin, astragalosides and curzenone extracted from a variety of traditional Chinese herbs (e.g. *Astragali* radix, *Paeoniae* radix rubra, *Curcumae* rhizoma, *Bupleuri* radix and *Eupolyphaga*), the majority of which have a long history as remedies for the treatment of chronic liver disease. For example, the use of *Astragali* radix in Chinese Medicine as a tonic herb is used alone or in conjunction with other herbs for the treatment of liver diseases. Pharmacological and clinical studies have demonstrated its hepatoprotective and other beneficial effects (18,19). The primary components of *Astragali* radix, astragalosides, were found to significantly inhibit the progression of CCl_4_-induced hepatic fibrosis *in vivo* as well as inhibit the proliferation of TGF-β1-stimulated HSCs *in vitro* (20). Another herb, *Paeoniae* radix rubra, has also been considered to be potent for the treatment for liver diseases. *Paeoniae* radix rubra extracts have been reported to reduce CCl_4_-induced liver fibrosis in rats as well as platelet-derived growth factor-stimulated hepatic stellate cell migration (21).

APE was prepared from extracts of *Astragali* radix, *Paeoniae* radix rubra, *Curcumae* rhizoma, *Bupleuri* radix and *Eupolyphaga* in the standard ratio 30:30:15:12:10 as measured by crude herbal weight. Previous studies have demonstrated that APE had protective effects on chemically-induced, acute liver injury in mice through the inhibition oxidative stress. In order to further investigate the anti-fibrotic activity of APE, the present study was designed to characterize the effects and elucidate the mechanism of APE treatment on CCl_4_-induced liver fibrosis in rats.

## Materials and methods

### Preparation of APE

The following herbs were purchased from The Xi’an Chinese Medicine Corporation, (Xi’an, China): *Astragali* radix, *Paeoniae* radix rubra, *Curcumae* rhizoma, *Bupleuri* radix and *Eupolyphaga*. All herbs used were identified by Dr Genquan Qiu, a specialist in Traditional Chinese Herbal Medicine and contributing author of the present study. Herb samples were preserved in the specimen room of the Institute of Clinical Pharmacology at Xi’an Medical College (Xi’an, China). APE components of the five herbs were extracted and prepared as follows:

A total of 8.45 kg of dried, sliced crude herbs (*Astragali* radix, *Paeoniae* radix rubra, *Curcumae* rhizoma, *Bupleuri* radix and *Eupolyphaga*) were prepared at a standard ratio of 30:30:15:12:10. Samples were decocted three times using 80 l water at 95°C for 35 min each time. The decocted solution was filtered though a150-μm gauze (Abcam, Cambridge, UK) and the filtrate was then concentrated to a mass of 4.22 kg (density, 1.225) using a vacuum desiccator (5530000; Labconoco, Kansas City, MO, USA) at 70°C. The sediment was dried into power using a spray drier (WD645; Titanium Industries, Inc., Taipei, Taiwan) at 80–160°C. This process yielded 2.11 kg dry powder. It should be noted that in all cellular *in vitro* experiments, APE powder was dissolved in Hank’s solution at a ratio of 1:50 (H9494, Sigma-Aldrich).

### Animals and treatment groups

Male Sprague-Dawley rats were obtained from the Experimental Animal Center (Anhui Medical University, Hefei, China) and had a weight range of 160–200g. All animals were housed in plastic cages a room temperature of 22±1°C, relative humidity of 50±20% and under a 12-h light/dark cycle. The studies were performed in accordance with the guidelines for the humane treatment of animals as set forth by the Association of Laboratory Animal Sciences and the Center for Laboratory Animal Sciences at Anhui Medical University. This study was approved by the ethics committee of Anhui Medical University (2011-002 SCXK; Hefei, China)

Chronic liver injury was induced using CCl_4_ injections as previously described (22–24). Rats were divided at random into six groups (n=10 per group). The control subjects were allowed *ad libitum* access to food and water. All other groups were administered an intraperitoneal injection of 1.0 ml/kg CCl_4_ in an olive oil vehicle (2:3 v/v) mixture, twice a week for eight weeks. The positive control group was intragastrically (i.g.) administrated colchicine (0.1 mg/kg; C3915; Sigma-Aldrich, St. Louis, MO, USA), a clinically used treatment for acute gout and other immunological diseases (25). Treatment groups received daily i.g. doses of APE, with each group receiving either 1.3, 2.6 or 5.2 g/kg. Oral i.g. administration of either colchicine or APE occurred on the day prior to CCl_4_ treatment. All subjects were weighed once per week throughout the duration of the experiment and the drug dose was adjusted to the body weight for each administration.

The rats were anesthetized with diethyl ether and sacrificed via cervical dislocation following the final injection of CCl_4_. Blood samples were then collected from the abdominal aorta. Samples were centrifuged for ten minutes (3,000 × g, 4°C) and serum was collected and then frozen at −80°C. Following sacrificing of the animals, the liver, spleen and kidney were promptly removed and weighed. A portion of the liver was fixed with paraffin for histopathology and the remaining tissue was stored at −80°C until further use.

### Analysis of liver function

The serum activities of aspartate aminotransferase (AST) and alanine aminotransferase (ALT) were determined using spectrophotometry with an Olympus AU 600 Autoanalyzer (Alternative Biomedical Solutions, Dallas, Texas, USA) and commercially available alanine aminotransferase assay and aspartate aminotransferase assay kits (Jiancheng Institute of Biotechnology, Nanjing, China). All absorbances were read at 505 nm and the enzyme activity was calculated as U/l.

### Activity measurements of antioxidants, antioxidant enzyme activity and malondialdehyde (MDA)

Liver homogenate (10%, w/v) was prepared by homogenizing the liver tissue on ice in 150 mM Tris-HCl buffered saline (pH 7.2; Sigma-Aldrich) using a polytron homogenizer (PT3100D; Kinematical, Lucerne, Switzerland). A commercially available kit (Jiancheng Biological Engineering Research Institute, Nanjing, China) was used to determine the activities of SOD and GSH-Px according to the manufacturer’s instructions. Data are expressed as SOD U/mg protein and GSH-Px mg/g protein (26). MDA levels in liver tissues were determined using the thiobarbituric acid method, provided by a commercially available kit (Jiancheng Biological Engineering Research Institute) and measured according to the manufacturer’s instructions.

### Measurement of fibrotic markers and serum levels of TGF-β1

Levels of hexadecenoic acid (HA), laminin (LN) and procollagen type III (PCIII) were assayed using a radioimmunoassay method with a kit obtained from Beijing North Institute of Biotechnology (Beijing, China). Hydroxyproline (hyp) and TGF-β1 serum levels were determined using an ELISA method using a kit obtained from Sigma-Aldrich. All absorbances were read at 450 nm using a microplate reader (FlexStation 3; Molecular Devices, Sunnyvale, CA, USA).

### Histopathological evaluation

Liver tissue was removed immediately following the sacrification of the animals and fixed in 10% formalin, then embedded in paraffin. Hematoxylin and eosin (HE) staining (E4283; Sigma-Aldrich) was performed to measure the degree of liver injury, while Masson staining (HT15; Sigma-Aldrich) was used to detect collagen deposition. Each staining method was performed according to the manufacturer’s instructions. Histopathological evaluation was performed by an expert pathologist, blinded to the treatment identity of each tissue sample. The degree of liver fibrosis was categorized into five groups according to the following scoring system: 0, no fibrosis, normal liver and absence of fibrosis; I, fibrosis present (collagen fibers present that extend from the portal triad or central vein to the peripheral region); II, mild fibrosis (mild collagen fibers present with extension without compartment formation); III, moderate fibrosis (moderate collagen fibers present with a certain level of pseudo-lobe formation); or IV, severe fibrosis (severe collagen fibers present with thickening of the partial compartments and frequent pseudo-lobe formation) (27). The degree of fibrosis was expressed as the mean of ten fields sampled from each slide. The final numerical score was calculated as follows: The sum of the number per grade of affected rats, divided by the total number of samples.

### Immunoblot analysis

Liver tissue samples were homogenized in extraction buffer (25 mmol 4-(2-hydroxyethyl)-1-piperazineethanesulfonic acid, 400 mmol KCl, 1 mmol EDTA and 1.5 mmol MgCl_2_) supplemented with protease and phosphatase inhibitors (Sigma-Aldrich) (1 mmol/l phenylmethyl sulfonyl fluoride, 0.1 mmol/l *N*-tosyl-L-phenylalanine chloromethyl ketone, 1 mg/ml aprotinin, 1 mg/ml pepstatin, 0.5 g/ml leupeptin, 1 mmol/l NaF, 1 mmol/l Na_4_P_2_O_4_ and 2 mmol/l Na_3_VO_4_). The extract was centrifuged at 14,000 × g for 10 min at 4°C and resulting supernatants were boiled for five min in SDS sample buffer [100 mmol/l Tris-HCl (pH 6.8), 4% SDS, 12% β-mercaptoethanol, 20% glycerol and 0.01% bromophenol blue] (Sigma-Aldrich). Samples were subjected to SDS-PAGE and transferred onto polyvinylidene difluoride (PVDF) membranes (Millipore, Bedford, MA, USA). The membrane was blocked overnight at 4°C by immersing in TBST-20 buffer, containing 10 mmol/l Tris-HCl, 150 mmol/l NaCl, 0.08% Tween 20 and 10% non-fat dry milk. PVDF membranes were then incubated with primary antibodies overnight at 4°C, followed by the appropriate secondary antibody for 2 h at room temperature. The primary antibodies used were as follows: Mouse anti-human monoclonal antibodies against collagen types I and III were purchased from Abcam (CAT: ab6308 and ab6310, Cambridge, UK), mouse anti-human monoclonal antibodies against TGF-β1 (CAT: sc-130348), mouse anti-Smad7 antibody (CAT: sc-365846) and rabbit/mouse anti-β-actin antibody (CAT: sc-7210 and sc-8432) were purchased from Santa Cruz Biotechnology (Santa Cruz, CA, USA). Rabbit anti-human phospho-Smad2 antibody (CAT: BS4172) and rabbit antihuman Smad2 antibody (CAT: BS2993) were purchased from Bioworld Technology (St. Louis, MO, USA). Rabbit anti-human phospho-Smad3 antibody (CAT: 9520), rabbit anti-human Smad3 antibody (CAT: 9513), rabbit anti-Smad4 (CAT: 9515) and rabbit anti-α-SMA antibody (CAT: A2522) were purchased from Cell Signaling Technology (Boston, MA, USA). The secondary antibodies used were goat anti-rabbit IgG and goat anti-mouse IgG (43413; 42472; Sigma-Aldrich).

The membranes were washed three times with TBST-20 buffer, immunoreactive proteins were detected using enhanced chemiluminescence medium (RPN 2106; Amersham Pharmacia Biotech, Piscataway, NJ, USA) and visualized by autoradiography (Bio-Rad, Hercules, CA, USA). Blots were then quantified via densitometric analysis using Quantity One version 4.52 software and a GelDoc XR (Bio-Rad).

### Statistical analyses

Quantitative data are expressed as mean ± standard deviation. The Student’s t-test was used to compare between groups and the Mann-Whitney rank sum test was used for the degree of histopathological liver injury. All tests were performed using SPSS version 13.0 software (SPSS, Inc., Chicago, IL, USA). P<0.05 was considered to indicate a statistically significant difference between values.

## Results

### APE mitigates the decrease in body weight induced by CCl_4_ treatment

**Figure 1:**
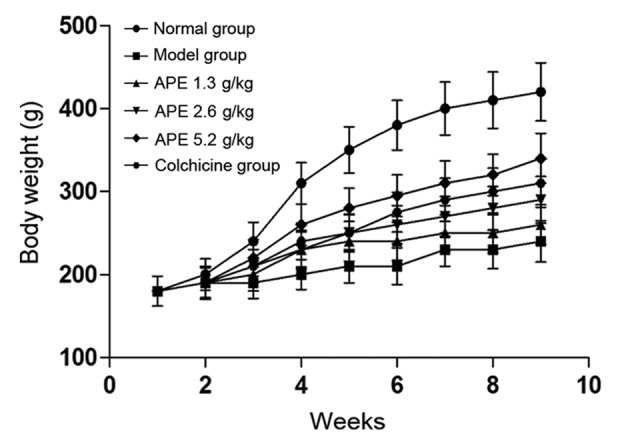
Body weight of rats from normal, model and drug-treated groups. The weights of rats in the model and CCl_4_ treament groups were markedly decreased when compared to those of the control group (P<0.05). When compared to the model group, APE (2.6 and 5.2 g/kg) administration significantly increased body weight in a dose-dependent manner (P<0.05). Low doses of APE (1.3 g/kg) and positive control (colchicine, 0.1 mg/kg) had no significant effect on the body weight of the animals (P>0.05). APE, *Astragalus* and *Paeoniae* radix rubra extract.

In comparison to the normal group, the weight of rats in the model group and the CCl_4_-treated groups was markedly decreased (P<0.05) (Fig. 1). The weight of animals receiving APE treatments (2.6 and 5.2 g/kg) significantly increased in a dose-dependent manner (P<0.05), compared to that of the model group. However, the lowest APE dose (1.3 g/kg) and colchicine (0.1 mg/kg), used as a positive control, had no significant effect on ameliorating CCl_4_-induced weight loss (P>0.05).

### APE treatment protects against CCl_4_-induced increases in liver and spleen coefficients, but not kidney coefficients

Liver, spleen and kidney coefficients were compared to organ coefficients obtained from rats with CCl_4_-induced fibrosis. As shown in Table I, the liver and spleen coefficients were significantly increased when compared to those of the control groups. More specifically, treatment with higher doses of APE (2.6 and 5.2 g/kg) markedly reduced the liver and spleen coefficients following CCl_4_ treatment (P<0.05). In the positive control group, colchicine (0.1 mg/kg) also reduced the liver coefficient (P<0.05); however, it showed a protective effect against the increased spleen coefficient following CCl_4_ treatment (P>0.05). No significant differences were observed in kidney coefficients among all treatment groups when compared to those of the controls (Table I).

### APE treatment mitigates CCl_4_-induced increases in serum AST and ALT levels

Serum ALT and AST activities were evaluated in order to measure the extent of liver injury following chronic CCl_4_ treatment. Serum levels of ALT and AST post-CCl_4_ treatment were increased three and four-fold, respectively, when compared to those of the control group (P<0.01) (Table II). Of note, high doses of APE (2.6 and 5.2 g/kg) and colchicine (0.1 mg/kg) demonstrated a significantly protective effect against the increase of serum ALT and AST following long-term treatment with CCl_4_ (P<0.05) (Table II).

### Effect of APE on levels of MDA, SOD and GSH-Px in hepatic tissue

GSH-Px and SOD have been reported to be capable of scavenging the toxic CCl_4_ metabolite lipid peroxide (28); therefore, levels of GSH-Px and SOD were measured in hepatic tissue samples from rats in order to determine the effect of APE on these compounds. As shown in Table III, the levels of GSH-Px and SOD were significantly decreased in the model group compared with those of the control group (P<0.01). Treatment with high doses of APE (2.6 and 5.2 g/kg) and colchicine (0.1 mg/kg) mitigated CCl_4_-induced GSH-Px and SOD depletion (P<0.05) (Table III). Following treatment with low doses of APE (1.3 g/kg), levels of GSH-Px and SOD were slightly, but not significantly, higher than those of animals administered CCl_4_ only.

In addition, MDA, a marker for lipid peroxidation levels, was significantly increased in rats with hepatic fibrosis compared with those of the model group (P<0.01). Of note, APE treatment (2.6 and 5.2 g/kg) resulted in an observable decrease in MDA levels (P<0.05). However, the colchicine group (0.1 mg/kg) showed no significant difference in MDA levels when compared with those of the model group (Table III).

### APE administration decreases serum HA, LN and PCIII and hydroxyproline levels in CCl_4_-induced fibrotic livers

As shown in Table IV, serum levels of three markers of liver fibrosis, HA, LN and PC III, were markedly increased in hepatic fibrotic rats when compared to those of the control group (P<0.01) (Table IV). Administration of APE (2.6 and 5.2 g/kg) or colchicine (0.1 mg/kg) effectively decreased CCl_4_-induced serum levels of HA, LN and PC III (P<0.05; P<0.01) (Table IV).

### APE reduces hapatic hydroxyproline, an index of liver fibrosis

Following long-term treatment with CCl_4_, rats were found to have increased levels of hydroxyproline when compared with those of the control group (P<0.01) (Table II). Treatment with APE (2.6 and 5.2 g/kg) resulted in significantly reduced levels of hydroxyproline in liver tissue when compared with those of the model group (P<0.05; P<0.01) (Table II). Colchicine (0.1 mg/kg) had an identical effect to that of high doses of APE on hydroxyproline levels (P<0.05) (Table II).

### APE results in improved liver histopathology

**Figure 2:**
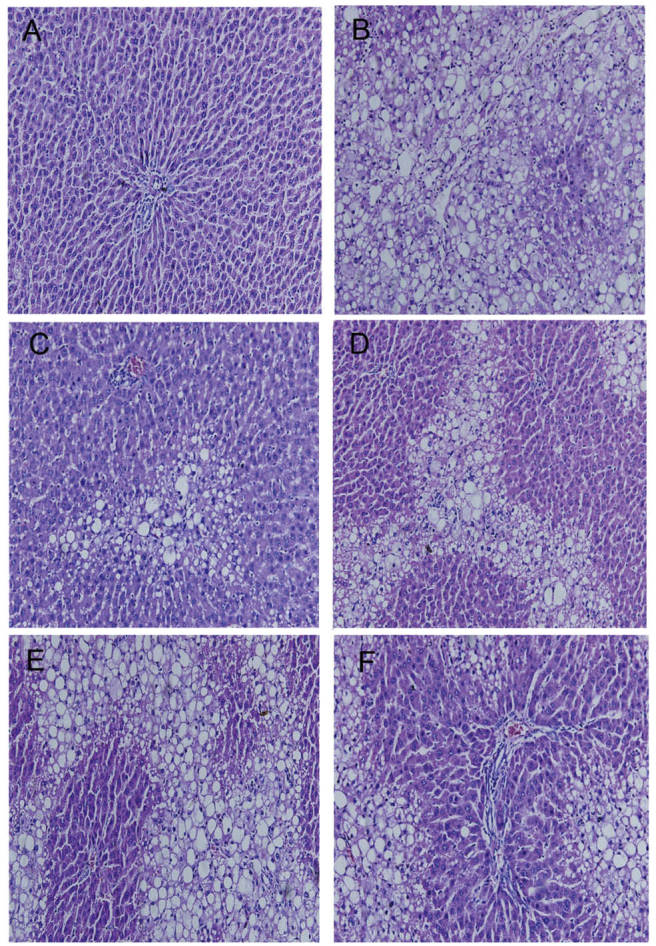
Effect of APE on the tissue morphology of fibrotic rat liver as indicated by hematoxylin and eosin staining (magnification, ×100). (A) Normal group, liver sections showed normal hepatic cells with well preserved cytoplasm, prominent nucleolus and central vein. (B) Model group, liver sections showed fatty acid degeneration, necrosis, hepatocyte ballooning and inflammatory cells infiltration. (C) APE group (5.2 g/kg); (D) APE group (2.6 g/kg); and (E) APE group (1.3 g/kg). Liver sections showed a dose-depenent reduction in the degree of liver injury and inflammation. (F) Colchicine 0.1 mg/kg, liver section showed a less severe degree of liver injury. APE, *Astragalus* and *Paeoniae* radix rubra extract.

HE staining indicated that CCl_4_ administration resulted in extensive morphological changes to the animals’ livers, including marked fatty acid degeneration, necrosis, hepatocyte ballooning and infiltration of inflammatory cells into the interstitial space of the livers. Liver tissue from the control group tissue showed normal lobular architecture with central veins and radiating hepatic cords (Fig. 2A). The histological pattern of tissue of subjects treated with APE (1.3, 2.6 and 5.2 g/kg) showed a lesser degree of liver injury and inflammation (Fig. 2C–E). A similar trend of reduced hepatic injury and inflammation was also observed in the tissue of colchicine-treated rats (Fig. 2F).

**Figure 3:**
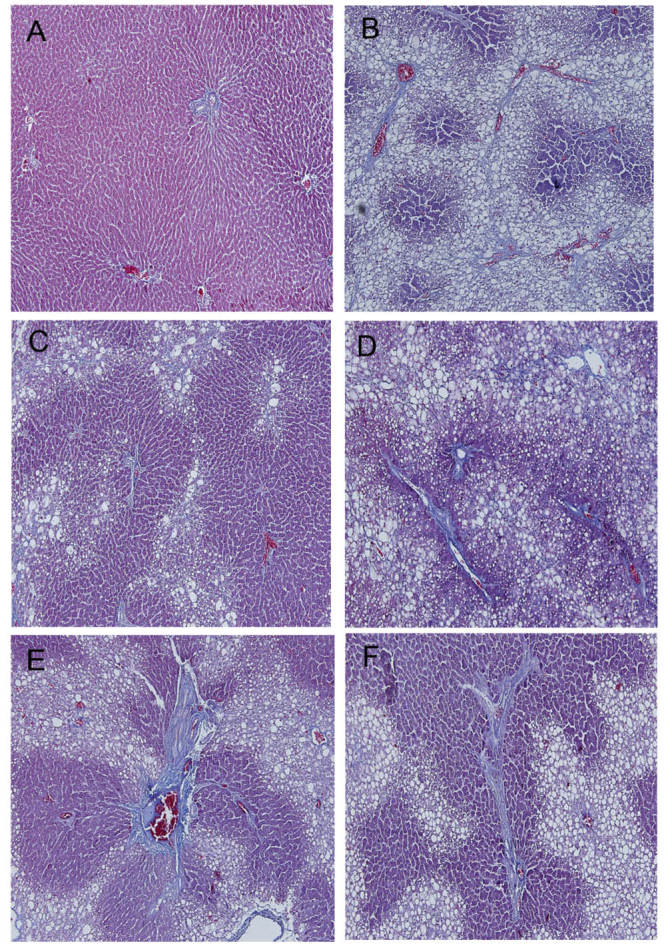
Effect of APE on the tissue morphology of fibrotic rat liver as indicated by Masson staining (magnification, ×100). (A) Normal group. (B) Model group, the livers of rats treated with CCl_4_ showed extensive accumulation of connective tissue resulting in the formation of continuous fibrotic septa, nodules of regeneration and noticeable alterations in the central vein as compared to the normal control. (C) APE group (5.2 g/kg); (D) APE group (2.6 g/kg); (E) APE group (1.3 g/kg); and (F) Colchicine 0.1 mg/kg. The groups with APE (1.3, 2.6 and 5.2 g/kg) and colchicine (0.1 mg/kg) showed less collagen deposited and pseudolobule formation. APE, *Astragalus* and *Paeoniae* radix rubra extract.

Masson staining was used to detect histopathological changes that resulted from CCl_4_-induced fibrosis (Fig. 3). The livers of rats treated with CCl_4_ showed extensive accumulation of connective tissues resulting in the formation of continuous fibrotic septa, nodules of regeneration and noticeable alterations to the central vein, compared to those of the healthy controls (Fig. 3A and B). APE- (1.3, 2.6 and 5.2 g/kg), and colchicine- (0.1 mg/kg) treated tissues showed reduced collagen deposition and fewer pseudolobule formation (Fig. 3C–F). Microscopic examination revealed that APE markedly reduced the grade of liver fibrosis and improved CCl_4_-induced hepatic fibrosis (P<0.05) (Table V).

### High doses of APE reduce TGF-β1 levels in rats with hepatic fibrosis

**Figure 4:**
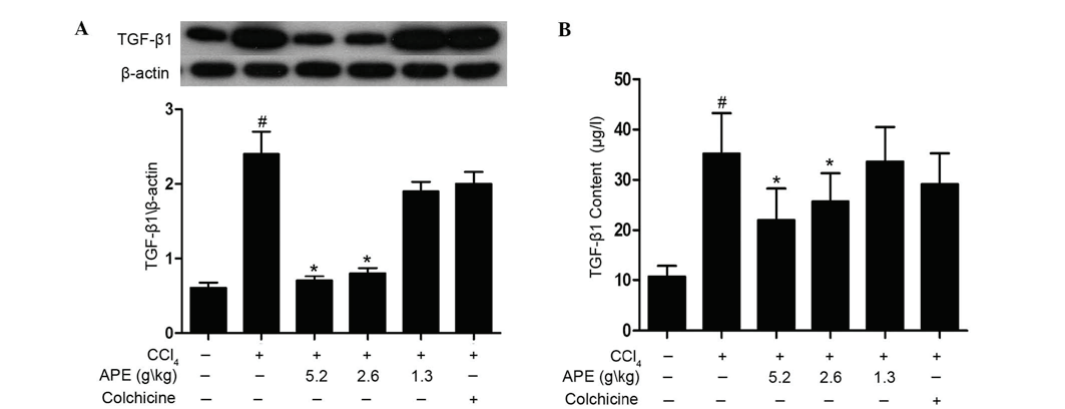
Effect of APE on production of TGF-β1 in livers of rats with hepatic fibrosis. (A) Western blot analysis indicated that APE treatment (2.6 and 5.2 g/kg) significantly decreased TGF-β1 expression levels in serum. (B) ELISA assay demonstrated that APE treatment (2.6 and 5.2 g/kg) notably reduced the levels of TGF-β1 in serum. ^#^P<0.05, TGF-β1 levels as compared to CCl_4_ control group; ^*^P<0.05, compared to model group. APE, *Astragalus* and *Paeoniae* radix rubra extract; TGF-β1, transforming growth factor β1.

The production of TGF-β1 was investigated using western blot analysis and ELISA assay. As shown in Fig. 4A, western blot analysis revealed that levels of TGF-β1 in hepatic fibrosis tissue were significantly elevated when compared to those of the normal control group (P<0.05). In addition, APE treatment (2.6 and 5.2 g/kg) significantly decreased TGF-β1 levels when compared to those of the CCl_4_-only control group (P<0.05). However, the low-dose APE group (1.3 g/kg) and colchicine (0.1 mg/kg) group showed no statistically significant differences to the CCl_4_-only control group. The ELISA assay also revealed that APE treatment (2.6 and 5.2 g/kg) reduced levels of TGF-β1 in serum (P<0.05) (Fig. 4B). These results indicated that high doses of APE (2.6 and 5.2 g/kg) have the capacity to mitigate increased levels of TGF-β1 in rats with CCl_4_-induced hepatic fibrosis.

### APE decreases Smad2/3 phosphorylation and Smad7 expression in the liver tissue of rats with CCl_4_-induced hepatic fibrosis

**Figure 5:**
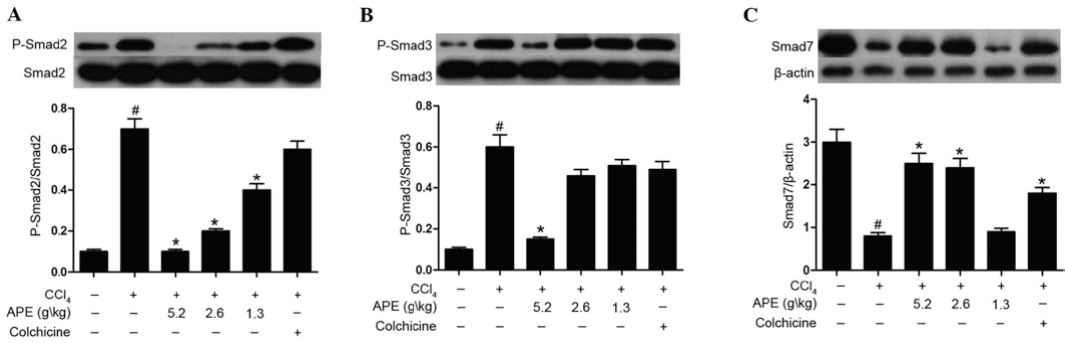
Effect of APE on Smad2/3 phosphorylation and Smad7 expression in livers of rats with CCl_4_-induced hepatic fibrosis. APE treatment (5.2 g/kg) significantly suppressed (A) Smad2 and (B) Smad3 phosphorylation in hepatic fibrosis tissue. APE treatment (1.3 and 2.6 g/kg) downregulated the phosphorylation of Smad2, but had no significant effect on Smad3 phosphorylation. (C) APE treatment (2.6 and 5.2 g/kg) and colchicine treatment (0.1 mg/kg) significantly elevated levels of Smad7 expression. ^#^P<0.05, compared to control group; ^*^P<0.05, compared to model group. APE, *Astragalus* and *Paeoniae* radix rubra extract.

As shown in Fig. 5, CCl_4_-induced hepatic fibrosis caused increased phosphorylation of Smad2 and Smad3 when compared to that of the controls. APE treatment (5.2 g/kg) significantly decreased Smad2 and Smad3 phosphorylation in liver tissue of rats with hepatic fibrosis (P<0.05). Lower doses of APE (1.3 and 2.6 g/kg) also downregulated the phosphorylation of Smad2, but had no significant effect on Smad3 phosphorylation (P<0.05). Similarly, colchicine treatment (0.1 mg/kg) had no significant effect on the phosphorylation of Smad2 or Smad3 (Fig. 5A and B).

Levels of Smad7 expression in the model group were markedly suppressed compared to those of the normal group (P<0.05). By contrast, APE (2.6 and 5.2 g/kg) and colchicine treatments (0.1 mg/kg) significantly increased levels of Smad7 expression compared to those of the model group (P<0.05).

In conclusion, these results revealed that high doses of APE (2.6 and 5.2 g/kg) prevented CCl_4_-induced fibrosis. The results also indicated that the mechanism of action of APE proceeded through preventing the phosphorylation of Smad2/3 and enhancing the inhibition of Smad7 expression in the TGF-β/Smad signaling pathway.

### APE decreases α-SAM, collagen I and collagen III expression in liver tissue of rates with CCl_4_-induced hepatic fibrosis

**Figure 6:**
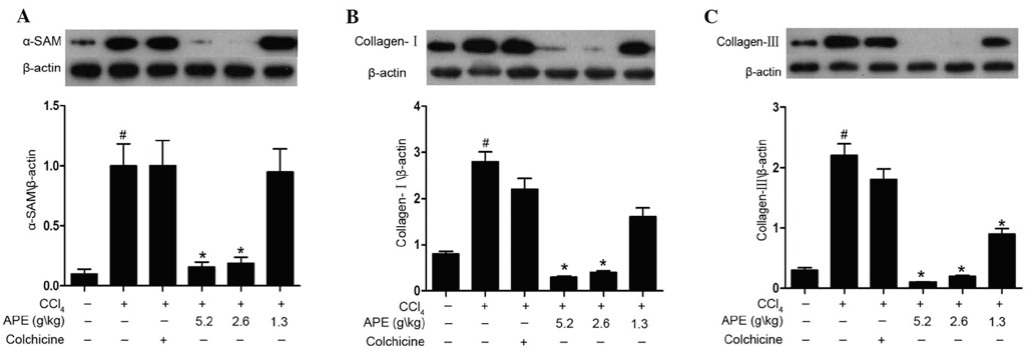
Effect of APE on α-SAM, collagen I and collagen III expression in livers of rats with CCl_4_-induced hepatic fibrosis. APE treatment (2.6 and 5.2 g/kg) decreased (A) α-SAM, (B) collagen I and (C) collagen III expression. APE treatment (1.3 g/kg) also downregulated collagen III expression. ^#^P<0.05, compared to control group; ^*^P<0.05, compared to model group. APE, *Astragalus* and *Paeoniae* radix rubra extract; α-SAM, α-smooth muscle actin.

Western blot analysis revealed increased protein expression of α-SAM, collagen I and collagen III in CCl_4_-induced hepatic fibrotic tissue when compared to expression levels in normal control rats (Fig. 6). APE treatment (2.6 and 5.2 g/kg) significantly decreased α-SAM, collagen I and collagen III expression compared to expression levels in the model group. Moreover, the low dose of APE (1.3 g/kg) was also able to decrease collagen III expression (P<0.05). However, no significant reductions were observed following treatment with colchicine (0.1 mg/kg).

## Discussion

It is well documented that HF is a common feature in numerous chronic hepatic diseases and that constant fibrosis may lead to the development of hepatocellular carcinoma (29). However, HF is only a small part of a dynamic cascade that begins with hepatocyte necrosis. Following necrosis, HSCs are activated and proliferate, with the concurrent release of fibrogenic transmitters or factors (30). However, studies have demonstrated that interrupting or reversing the molecular pathways involved in hepatic fibrosis may be promising for future therapies (31,32). Previous studies have shown that APE had protective effects against acute liver injury in murine models. To further investigate the effects of APE on liver fibrosis, the present study used the well-characterized CCl_4_-induced hepatic fibrosis model (33). Liver injury has been reported to induce the release of two aminotransferases, AST and ALT, into the circulatory system (34). These proteins are therefore used as conventional indicators of hepatic trauma. Throughout the present study, body weight, organ coefficients and the levels of ALT and AST were assessed to evaluate the extent and degree of liver injury in rats undergoing long-term CCl_4_ treatment. The results indicated that high doses of APE (2.6 and 5.2 g/kg) reduced inflammation and alleviated CCl_4_-induced liver injury. In addition, histopathological examinations using HE staining provided further evidence for the hepatoprotective effect of APE in rats.

HF is characterized by excessive deposition of ECM components, including collagen protein, proteoglycan and osamine protein (35). Therefore, administration of APE may protect the liver against hepatic injury by reducing collagen deposition. HA, LN and PC III are essential components of the ECM and, due to their abnormally increased expression in liver fibrosis, are conducive biomarkers for hepatic fibrogenesis (36,37). Increased expression of hepatic hyp is another liver index that represents the degree of HF (38). In the present study, the levels of serum HA, LN, PC III and the content of hyp in hepatic tissue were significantly increased in CCl_4_-treated rats, whereas APE treatment (2.6 and 5.2 g/kg) markedly decreased the levels of serum HA, LN, PC III and the content of hyp in hepatic tissue. These significant results strongly suggested the presence of inherent hepatoprotective effects of APE. Furthermore, histopathological observations provided additional confirmation that the severity of CCl_4_-induced liver fibrosis was largely ameliorated by treatment with APE.

Oxidative stress has an important role in the generation of CCl_4_-induced liver fibrosis (39). CCl_4_ damages the hepatocellular membrane via lipid peroxidation, which is followed by the release of inflammatory mediators from activated inflammatory cells. The release of these mediators is thought to potentiate CCl_4_-induced hepatic injury (28). Numerous antioxidants have been shown to have therapeutic or protective effects against liver injury (40,41). The concentration of the main product of lipid peroxidation, MDA, is generally assayed as the total levels of lipid peroxidation products (42). SOD and GSH-Px are key enzymes in the antioxidant defense system and their mechanism of action involes catalysis of the transformation of hydrogen peroxide into water (43). In the present study, CCl_4_-treated rats demonstrated elevated levels of MDA and decreased activity of SOD and GSH-Px. However, treatment with APE (2.6 and 5.2 g/kg) reduced the amount of MDA and increased the activities of SOD and GSH-Px. This therefore indicates that APE may have an anti-oxidative role in hepatic fibrosis through recovery of the organism’s natural anti-oxidative defense system.

HSCs are a major type of fibrogenic liver cell found during liver injury and have are responsible for the progression of hepatic fibrosis (44). TGF-β1 has been suggested to be an important factor in activating and promoting the transformation of HSCs (45). The present study demonstrated that TGF-β1 was highly expressed in CCl_4_-treated rats and administration of APE resulted in a significant reduction of TGF-β1 levels.

TGF-β1 controls a diverse set of cellular processes and its canonical signaling is mediated via TGF-β-induced phosphorylation of receptor-activated Smad2 and Smad3 (46). Smad7 is the negative feedback regulator for TGF-β signaling, acting to antagonize the activity of the receptor-regulated Smads, which leads to the termination of TGF-β signaling (47). The present study showed that APE significantly inhibited the phosphorylation of Smad2 and Smad3 and reversed the inhibitory effect of CCl_4_ treatment on Smad7 expression. Therefore, it was hypothesized that the anti-fibrotic effects of APE occur via the TGF-β1/Smad pathway.

Following long-term CCl_4_-stimulation, HSCs become activated and transdifferentiate into myofibroblasts (MFBs). MFBs are characterized by numerous fibrotic functions, including the induction of ECM deposition, α-SMA expression as well as the synthesis and secretion of type I and type III collagen (48). Western blot analysis performed in the present study revealed that CCl_4_ enhanced the expression of α-SMA, type I and type III collagen, while the administration of APE prevented fibrosis development through inhibition of HSCs.

In conclusion, the present study demonstrated that APE was effective in preventing necro-inflammation and fibrogenesis in CCl_4_-induced hepatic fibrosis. Of note, the hepatoprotective effects of APE appear to be associated with the inhibition of lipid peroxidation and decreases in TGF-β1 levels. However, further studies are required to determine whether APE exerts these protective effects in human subjects *in vivo* and whether it has an effect on other types of fibrogenesis. In addition, the exact mechanism and pharmacological actions of APE for clinical usage remain to be elucidated.

## Figures and Tables

**Table I:** Effect of APE on organ coefficients in rats with CCl_4_-induced hepatic fibrosis (n=10).

Groups	Dose (g/kg)	Liver coefficient (%)	Spleen coefficient (%)	Kidney coefficient (%)
Normal	—	2.64±0.22	0.24±0.06	0.68±0.07
Model	—	4.65±0.41^a^	0.38±0.07^a^	0.69±0.11
APE	1.3	4.33±0.45	0.34±0.05	0.69±0.08
	2.6	4.21±0.40^b^	0.31±0.05^b^	0.65±0.13
	5.2	4.13±0.38^c^	0.30±0.04^c^	0.67±0.11
Colchicine	0.1	4.26±0.37^b^	0.32±0.06	0.58±0.09

Values are expressed as the mean ± standard deviation;

a P<0.01 compared with control group;

b P<0.05,

c P<0.01 compared with model group.

APE, *Astragalus* and *Paeoniae* radix rubra.

**Table II:** Effect of APE on liver function, hydroxyproline content and production of TGF-β1 in rats with CCl_4_-induced hepatic fibrosis (n=10).

Group	Dose (mg/kg)	ALT (U/l)	AST (U/l)	Hydroxyproline (mg/g)
Normal	—	42.62±10.36	36.49±9.88	1.08±0.29
Model	—	126.53±31.59^a^	116.93±28.47^a^	2.96±0.85^a^
APE	1.3	109.48±20.86	98.86.46±18.53	2.54±0.58
	2.6	91.42±18.28^b^	89.36±19.48^b^	2.18±0.48^b^
	5.2	86.94±20.84^b^	81.75±18.47^b^	2.08±0.36^c^
Colchicine	0.1	96.58±18.93^b^	90.86±21.61^b^	2.24±0.34^b^

Values are expressed as the mean ± standard deviation;

a P<0.01 compared with control group;

b P<0.05,

c P<0.01 compared with model group.

APE, *Astragalus* and *Paeoniae* radix rubra; TGF-β1, transforming growth factor-β1; ALT, alanine aminotransferase; AST, aminotransferase.

**Table III:** Effect of APE on MDA levels, and SOD and GSH-Px activities in liver homogenates of CCl_4_-induced liver fibrosis in rats (n=10).

Group	Dose (g/kg)	GSH-Px (U/mg)	SOD (U/mg)	MDA (μmol/g)
Normal	—	397.85±54.58	247.62±41.82	42.16±9.86
Model	—	215.51±42.43^a^	146.83±28.68^a^	71.83±11.35^a^
APE	1.3	246.25±31.46	161.52±30.49	62.83±12.63
	2.6	265.31±32.46^b^	183.64±29.34^b^	56.83±12.64^b^
	5.2	297.61±34.56^c^	195.39±32.94^b^	52.61±13.48^b^
Colchicine	0.1	271.68±31.83^b^	174.22±28.57^b^	58.23±15.61

Values are expressed as the mean ± standard deviation.

a P<0.01 compared with control group;

b P<0.05,

c P<0.01 compared with model group.

APE, *Astragalus* and *Paeoniae* radix rubra extract; GSH-Px, glutathione; SOD, superoxide dismutase; MDA, malondialdehyde.

**Table IV:** Effect of APE on serum HA, LN and PCIII levels in rats with CCl_4_-induced liver fibrosis (n=10).

Group	Dose (mg/kg)	HA (μg/l)	LN (μg/l)	PCIII (μg/l)
Normal	—	106.38±27.69	116.95±36.56	108.62±28.64
Model	—	256.83±52.68^a^	297.62±48.31^a^	286.34±45.29^a^
APE	1.3	221.96±38.94	264.83±41.66	257.34±46.92
	2.6	208.53±38.42^b^	238.18±38.71^b^	228.67±42.31^b^
	5.2	195.21±39.48^c^	224.73±34.16^c^	208.71±43.53^c^
Colchicine	0.1	211.62±37.49^c^	240.68±46.48^a^	248.57±42.16^b^

Values are expressed as the mean ± standard deviation;

a P<0.01 compared with control group;

b P<0.05,

c P<0.01 compared with model group.

APE, *Astragalus* and *Paeoniae* radix rubra extract; HA, hexadecenoic acid; LN, laminin; PCIII, procollagen type III.

**Table V:** Effect of APE on the pathological grading of CCl_4_-induced liver fibrosis in rats.

		Pathological grading of hepatic fibrosis					
			
Group	Dose (mg/kg)	0	I	II	III	IV	P-value
Normal	—	10	0	0	0	0	—
Model	—	0	0	1	4	6	0.000^a^
APE	1.3	0	1	2	3	4	0.467
	2.6	0	2	4	2	2	0.031^b^
	5.2	1	4	2	3	0	0.014^b^
Colchicine	0.1	0	1	3	3	2	0.029^b^

Ten animals were used to obtain these results, which are presented as the mean of ten fields of view.

a P<0.01 compared with control group;

b P<0.05 compared with model group.

APE, *Astragalus* and *Paeoniae* radix rubra.
